# Are early pregnancies implanted close to the tubal ostia at increased risk of miscarriage? A prospective observational study

**DOI:** 10.1177/1742271X231225077

**Published:** 2024-03-10

**Authors:** Salwa Abdullahi Idle, Laura Ferrara, Archana Vasireddy, Katherine Andersen, Jemma Johns, James Barrett, Jackie A Ross

**Affiliations:** 1 School of Medical Education, King’s College London, London, UK; 2 King’s College London, London, UK; 3 King’s College Hospital NHS Foundation Trust, London, UK; 4 University College London, London, UK; 5 King’s College Hospital, London, UK

**Keywords:** Early pregnancy, eccentric pregnancy, lateral pregnancy, angular

## Abstract

**Introduction:**

The aim of this article is to assess any association between the risk of miscarriage and the distance of an early pregnancy from the closest tubal ostia.

**Methods:**

Early pregnancy was defined as a gestational sac ⩽ 15 mm mean diameter within the upper half of the endometrial cavity. The shortest distance from the gestational sac (chorionic membrane) to the closest tubal ostia and the interostial distance were measured. The outcomes for pregnancies at varying distances from a tubal ostia were compared using Fisher’s exact test. The receiver operating characteristic curve assessed the distance from the sac to the ostia as a predictor of miscarriage. A Wilcoxon rank-sum test was used to assess any difference in the distance from the ostia between women who miscarried and those who did not.

**Results:**

Outcome data were available for 212/230 patients. The relative risk of miscarriage was 5/6 (83%) in the group with gestational sacs ⩽ 4 mm from the ostium versus 70/206 (34%) > 4 mm (*p* = 0.02). The proportion of miscarriages was 11/20 (55%) if the distance from the tubal ostium was ⩽5 mm versus 64/192 (33%) if >5 mm (*p* = 0.08). There was a good neonatal outcome for those with live births.

**Conclusion:**

The risk of first trimester miscarriage was high with early pregnancies implanted close to the tubal ostia, but this finding did not reach statistical significance. A larger study is needed to establish whether 4 or 5 mm could be used as a clinically useful criterion for defining early pregnancies that are at increased risk of miscarriage.

## Introduction

The term ‘angular pregnancy’ (AP) has been used in the past to describe a pregnancy implanted within the uterine cavity but directly over the tubal opening.^1–3^ Prior to the introduction of imaging into medical practice, it was defined surgically as a pregnancy implanted within the lateral angle of the uterus, medial to the interstitial portion of the fallopian tube and displacing the reflection of the round ligament laterally.^2^ A higher prevalence of complications such as pelvic pain, vaginal bleeding, tendency to miscarriage and increased risk of retained placenta after delivery have all been described in APs.^4–7^ This may well have been due to the difficulty in making an accurate diagnosis and distinguishing a complete or partial interstitial ectopic pregnancy from a laterally implanted eutopic pregnancy. However, even given this difficulty, the conclusion of Jansen and Elliot’s 1981 review^2^ was that APs should be managed expectantly in the presence of a firm surgical diagnosis, an ongoing pregnancy and healthy myometrium over the gestational sac (GS) because most continued normally.

It is debated whether the term ‘angular pregnancy’ should be used in modern practice.^8^ The differential diagnosis of a pregnancy implanted high and lateral within the uterus is between an interstitial ectopic pregnancy implanted more laterally, within the proximal fallopian tube (with almost inevitable rupture if the pregnancy is ongoing) and a normally sited pregnancy more medially.^9^ Distinguishing between the two can be particularly difficult with an arcuate-shaped uterus, if the endometrium is thin or if the endometrial cavity is scarred. The terms ‘interstitial’, ‘angular’ and ‘cornual’ with reference to pregnancy are often used improperly and inconsistently, generating confusion in counselling and management.^10–12^ It may be that ‘angular pregnancy’ is useful to retain as a term, but only if it defines a condition associated with a clinical outcome that is different to other implantation sites within the endometrial cavity.

Our aim was to prospectively study early intrauterine pregnancies implanted in the upper uterine cavity to assess whether there was any increased risk of miscarriage according to the laterality of the implantation site.

## Methods

This was a prospective observational study of women who attended the Early Pregnancy Unit at a tertiary hospital between December 2017 and October 2019. Women who had a pregnancy with a gestational sac diameter (GSD) of 2–15 mm with >50% of the GS located within the upper half of the uterine cavity and surrounded by endometrium on two-dimensional (2D) scan were eligible. All patients had an assessment by gynaecologists undergoing level 2 training in early pregnancy and gynaecological ultrasound using the same model of ultrasound equipment and a 7.5 MHz transvaginal probe (Voluson E8, GE Medical Systems, Milwaukee, WI, USA). We excluded women with pregnancies implanted in the lower half of the endometrial cavity. We also excluded women with pregnancies of unknown location, ectopic pregnancies, intention to terminate their pregnancy and larger GSD than specified in the inclusion criteria. Women with distorted uterine cavities due to fibroids or adenomyosis, which make difficult to measure the location using our pre-defined criteria, and didelphic or unicornuate uteri were also excluded.

**Figure 1.**
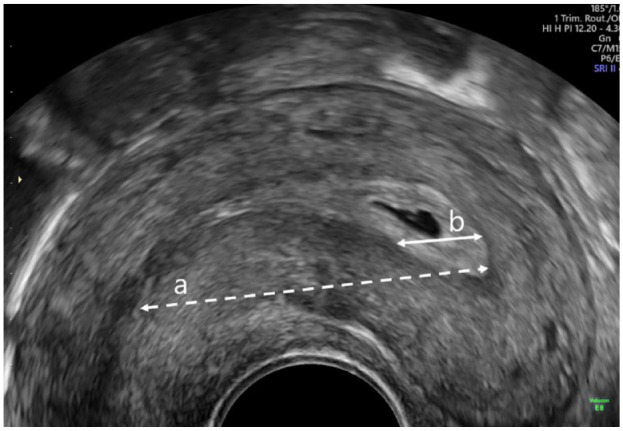
Image showing cavity length from the internal os to the fundus in sagittal section (a) and distance from the centre of the gestational sac to the fundus in sagittal or parasagittal section (b).

**Figure 2.**
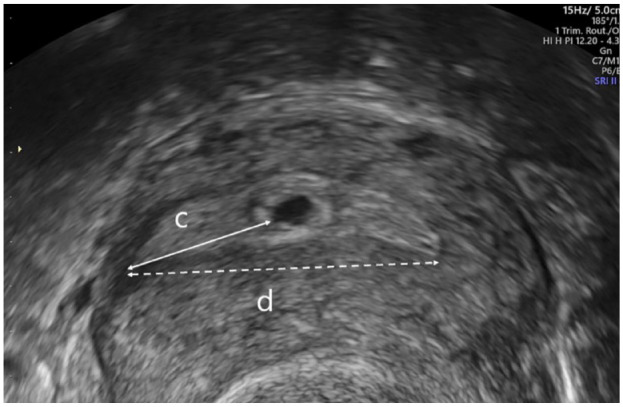
Image showing how to obtain measurement (c): distance from the chorionic membrane to the closest tubal ostia and (d): interostial distance.

Measurements recorded on 2D ultrasonography (US) were the estimated cavity length measured as a straight line from the internal os to the fundus in sagittal section (a) and distance from the centre of the GS to the fundus in sagittal or parasagittal section (b). Only women with pregnancies in the upper half of the cavity (b < a/2) were eligible (Figure 1). We also measured the distance from the chorionic membrane to the closest tubal ostia (c) and interostial distance (d) (Figure 2). A previous analysis by our study team showed the repeatability and reproducibility of these measurements to be reliable.^13^ The GSD, presence of a yolk sac (YS), an embryonic pole with measurements of crown-rump length (CRL) and heart rate (HR) were assessed and recorded as per routine clinical practice. Participants had a follow-up scan by the research team after 2 weeks to assess viability. Maternal data collected included presenting symptoms, age, ethnicity, body mass index (BMI), smoking and previous gynaecology and obstetric history.

Our primary study outcome measure was the progression of a pregnancy beyond 24 weeks gestation versus miscarriage. Our secondary outcome measure was the outcome of ongoing pregnancies. Outcomes were obtained from electronic patient records and by telephone, post, or email questionnaire. The gestation at delivery, any pregnancy complications, birthweight, and the presence of neonatal congenital anomaly were recorded.

## Statistical analysis

Our aim was to perform an exploratory analysis on data collected over a roughly 2-year period from early pregnancies implanted in the upper endometrial cavity to assess whether there was any evidence of an increased risk of miscarriage in those close to the tubal ostia. Anonymised data were transferred from the Viewpoint database (PIA Foetal Database, version 2.23; Viewpoint Bildverarbeitung GmbH, Munich, Germany) into Excel (Microsoft1 Office 2019) and statistical analyses were undertaken with SPSS and R (IBM Corp. Released 2020. IBM SPSS Statistics for Windows, Version 27.0. Armonk, NY: IBM Corp). Mean and standard deviation were used to describe continuous variables, and frequencies and percentages for categorical variables. A Wilcoxon rank-sum test with continuity correction was used to compare the distance of the GS from the tubal ostia between women who miscarried and those who did not. A receiver operating characteristic curve (ROC) was constructed to assess the relationship between the distance of the GS from the tubal ostia and the risk of miscarriage. The association between miscarriage and the distance of the GS from the tubal ostia (c) was assessed using Fisher’s exact test, for different values of c. Linear regression analysis was used to compare the primary outcome with respect to (c) while controlling for age, BMI, smoking, previous ectopic and previous surgery. Normalised values of c were normalised for the uterine cavity dimensions by dividing each value by $\sqrt{a^{2}+d^{2}}$ .

## Results

We recruited 230 women who attended our Early Pregnancy Unit and were eligible for the study. Of these, 10 subsequently chose to terminate their pregnancies for psycho-social reasons and 8 were lost to follow-up. This left 212 patients for analysis. The demographic and clinical data of the study group are shown in Table 1.

**Table 1.** Demographic and clinical characteristics of the study population.

Characteristic	*n* = 212	*p* value
Age (years), mean (SD)	31.5 ± 5.9	
BMI (kg/m^2^), mean (SD)	26.5 ± 5.7	0.8
Smoking	24 (11)	0.49
Ethnicity, *n* (%)		
Caucasian	101 (47.6)	
Black	73 (34.4)	
Asian	14 (6.6)	
Mixed	13 (6.1)	
Other	11 (5.1)	
Parity, *n* (%)		
Nulliparous	120 (56.6)	
>1	37 (17.5)	
Previous ectopic, *n* (%)	21 (9.9)	
Previous miscarriage, *n* (%)	88 (41.5)	
Recurrent miscarriage (>2)	9 (4.2)	
Conception, *n* (%)		
Planned	150 (70.8)	
Spontaneous	211 (99.5)	
Presentation, *n* (%)		
Abdominal pain	94 (44.3)	
Vaginal bleeding	81 (38.2)	
Other (including dating, reassurance, uncertain viability)	37 (17.5)	

SD: standard deviation; BMI: body mass index.

**Figure 3.**
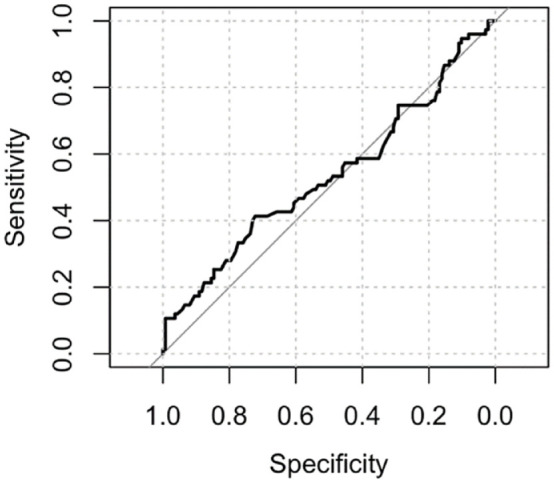
The ROC curve did not demonstrate an association between the proximity of a GSD to the ostia and the outcome of the pregnancy.

**Figure 4.**
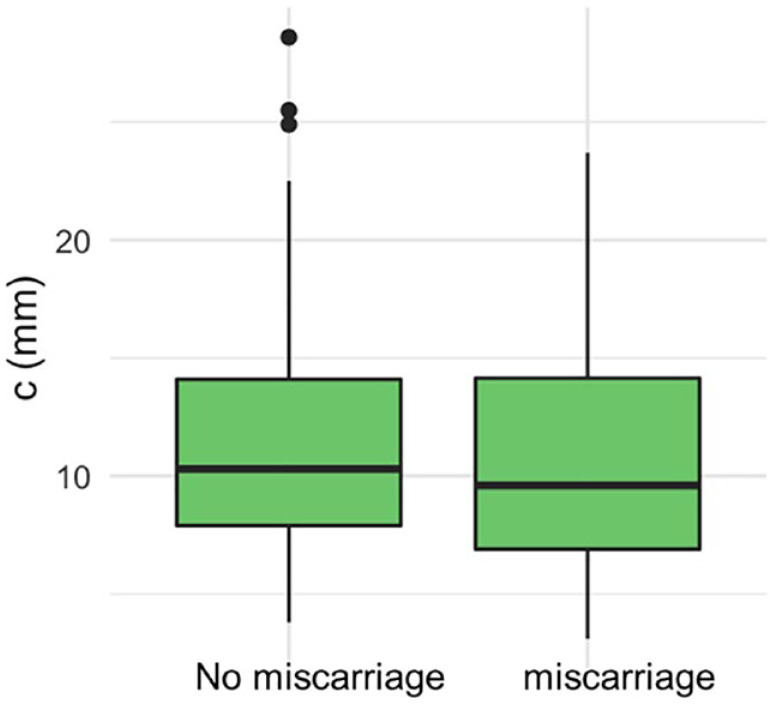
There was no difference in the distance of the gestational sac to the closest tubal ostia (c) between the women who miscarried and those who did not.

**Figure 5.**
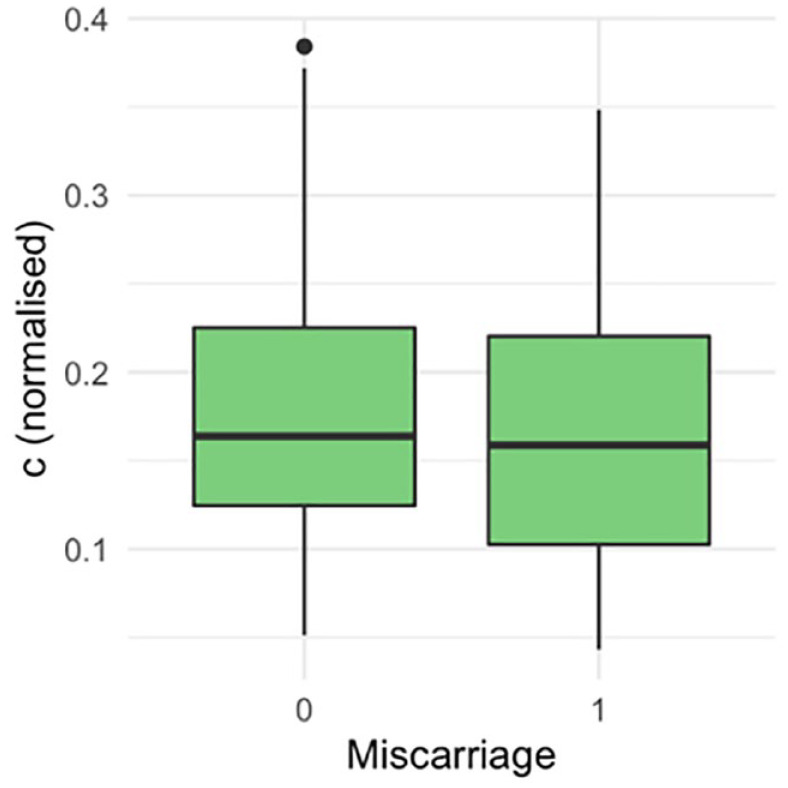
Computing the ‘hypotenuse’ of the uterus (*h* =√(*a*^2^ + *d*^2^) and dividing each value of c by the corresponding h to give a value for c relative to the size of the uterus.

**Figure 6.**
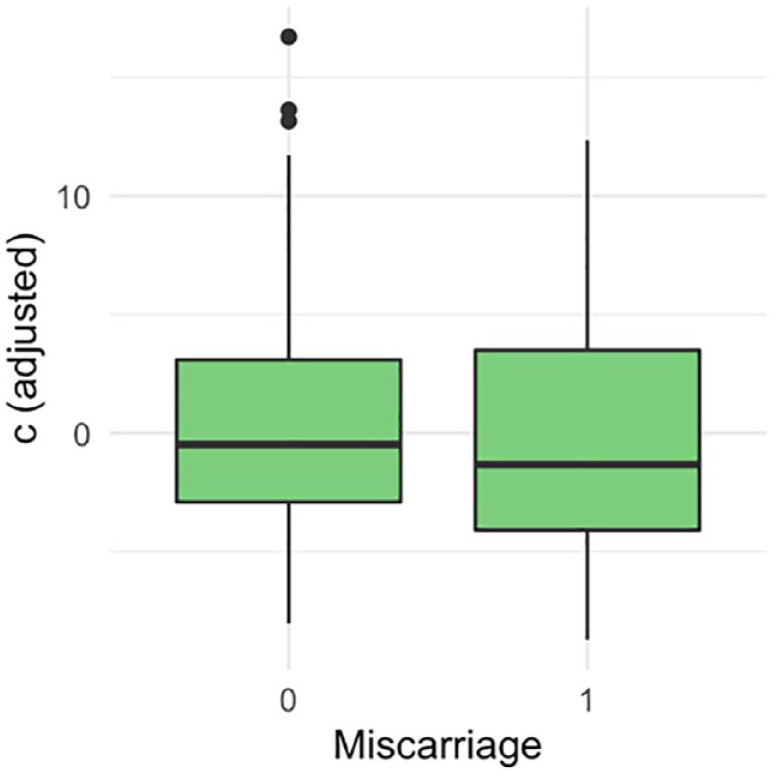
Linearly adjusting for the ‘hypotenuse’ h, and gestational sac diameter (GSD).

There were 136 (64%) livebirths, 75 (35.5%) miscarriages and 1 (0.5%) stillbirth. The ROC curve did not demonstrate a statistically significant association between the proximity of a GSD to the ostia and the outcome of the pregnancy (Figure 3). The area under the ROC curve was 0.54 (95% confidence interval (CI): 0.45–0.62). There was no statistically significant difference in the distance of the GS to the closest tubal ostia (c) between the women who miscarried and those who did not (Figure 4; *p* = 0.40). There was no statistically significant difference between the distance of the GS from the ostia (c) after normalising for the uterine cavity dimensions (Figure 5, *p* = 0.78) and GSD (Figure 6, *p* = 0.57).

We assessed whether there was a cut-off value of c associated with an increased risk of miscarriage (Table 2). Using incremental steps, we found that the risk of miscarriage was greatest in the group with c ⩽ 4 mm (5/6, 83% vs 70/206, 34%) and this was statistically significant (*p* = 0.02). We observed that the rate of first trimester miscarriage c ⩽ 5 mm 11/20 (55%) versus c > 5 mm, 64/192 (33%) did not quite reach statistical significance (*p* = 0.08). After correction for multiple testing, none of these findings were statistically significant. When comparing outcomes using regression analysis considering our clinical and ultrasound parameters, only maternal age was significantly associated with miscarriage (*p* = 0.02).

**Table 2.** Risk of miscarriage according to c.

c (mm)	Pregnancies, *n*	% pregnancies (95% CI)	Miscarriage (*n*)	% miscarrying (95% CI)	*p*
⩽4	6	3 (2–13)	5	83 (42–99)	0.0219
⩽5	20	20 (12–31)	11	55 (34–74)	0.0829
⩽6	33	15 (22–46)	16	48 (33–65)	0.1124
⩽7	47	22 (34–62)	21	45 (31–59)	0.1662
⩽8	67	31 (21–44)	30	45 (33–57)	0.0638
⩽9	91	43 (73–112)	34	37 (28–48)	0.5616
⩽10	105	50 (85–127)	38	36 (28–46)	0.7735
>10	107	50 (87–129)	36	34 (25–43)	0.7735

CI: confidence interval.

Considering secondary outcomes and c ⩽ 5 mm vs > 5 mm, women with a live birth delivered at a mean of 39 weeks gestation in both groups with comparable mode of deliveries; 78% vs 78% vaginal deliveries; 11% vs 16% emergency caesarean sections; and 11% vs 12% elective caesarean sections. There were 2 stillbirths (1.6%) recorded in the group c > 5 mm. Other pregnancy complications such as preterm birth, antepartum haemorrhage and placental complications were not found in the c ⩽ 5 mm and were 10% in the c > 5 mm group.

## Discussion

Our study has shown that there may be a higher risk of miscarriage in early pregnancies implanted high and laterally in the endometrial cavity, within 4 mm of a tubal ostium, although this was no longer significant after correction for multiple testing. Pregnancies within 5 mm had a 66% increase in the risk of miscarriage, with 55% miscarrying versus 33% of medially implanted pregnancies, although again, this difference did not reach statistical significance. Laterally implanted pregnancies did not appear to have a worse prognosis if the pregnancy continued beyond 24 weeks.

Pregnancies implanted high and lateral within the endometrial cavity have been termed ‘angular pregnancies’ and they have been reported in the literature over the past century.^1,14,15^ Despite transvaginal sonography being used routinely in the diagnosis of early pregnancy and early pregnancy complications for nearly 30 years, there are no standardised ultrasonic diagnostic criteria^4,16^ and it is debated whether the term should continue to be used. The current suggested criteria are a pregnancy in a non-anomalous uterus, less than 10 mm myometrial thickness from the GS to the outer border of the uterus, presence of endometrium completely surrounding the pregnancy and lack of an ‘interstitial sign’ (with a sensitivity of 80% and specificity of 98% for the diagnosis of interstitial ectopic pregnancy).^4,11,17,18^ We did not evaluate the myometrial thickness over the pregnancies as it has been shown to be a poor diagnostic sign. Yen et al. described an ‘angular pregnancy’ with a myometrial minimal thickness of 4.6 mm with a large uterine bulge on laparoscopy, whereas other cases with similar myometrial thickness had a normal laparoscopic appearance.^19,20^ In a recent case series of ‘angular pregnancies’, Bollig and Schust^18^ reported an average myometrial thickness of 5.1 ± 1.6 mm (95% CI = 4.6–5.6 mm) with 93% of follow-up scans showing no further thinning. The authors concluded that recording the pregnancy as eccentric is more important than strict limits of myometrial thickness.^18^ This group reported a live birth-rate of 80% with a 20% pregnancy failure rate,^18^ so their cohort of women with ‘angular’ pregnancies had better outcomes than our whole study population of women seen in our inner-city early pregnancy unit. Our pregnancy failure rate of 35% is likely a reflection of an earlier diagnosis of pregnancy, with average gestation at first scan at 5 + 2 weeks versus 7 + 4 weeks in Bollig’s study. There were no reports of adverse pregnancy complications such as placental adherence, uterine rupture or hysterectomy by Bollig’s team which is similar to the findings in our study.

Describing a normally located early pregnancy as ‘angular’ or ‘eccentric’ is only appropriate if it is clinically relevant. Otherwise, it risks causing unwarranted anxiety and unnecessary examinations. An important aspect of our study was the detailed examination of a GS in the lateral intrauterine cavity and defining the location using prespecified measurements which has not been done before. Constructing the coronal plane by three-dimensional (3D) ultrasound has assisted in the diagnosis of an AP in previous reported cases^10,21^ and the accuracy that 3D US offers in the diagnosis of uterine anomalies has been proven.^22,23^ However, we found that 2D US is more reliable for mapping early intrauterine pregnancies in our routine clinical practice.

A few papers have described ‘angular pregnancies’ as migrating, either by growing into the uterine cavity^18,24^ or the interstitial portion of the fallopian tube.^3,25,26^ However, an ‘angular’ pregnancy is not likely to grow laterally into the interstitial portion of the fallopian tube and become an ectopic pregnancy unless it were at least partially implanted there. The diagnosis of a partial interstitial ectopic pregnancy is a difficult one to make, highlighting the importance of visualising the endometrium surrounding the GS, excluding implantation in the interstitial tube. We propose that ‘angular’ pregnancies are normally located intrauterine pregnancies from the outset; they do not migrate but tend to grow medially towards the central portion of the endometrial cavity as the GS expands and the definitive placenta forms. It is biologically implausible that there is a threshold measurement of the distance from the tubal ostia that makes miscarriage inevitable or suddenly increases the risk of miscarriage, but it may be a factor in some pregnancies implanted with the villi over the embryonic pole close to the ostia, in the thinnest part of the uterus.

The strengths of this study are that this is a prospective observational study using predefined, validated ultrasound measurements to describe the location of early pregnancies in the upper aspect of the endometrial cavity. By restricting the study to only assessing very early pregnancies (GSD < 15 mm, approximating to 6 weeks gestation), we were able to measure the distance of the GS from the tubal ostium before the cavity was distorted by the expanding pregnancy. This allowed us to find suggested criteria to base a validation study on.

The limitation of the study is that it was underpowered, and the background risk of miscarriage was higher than expected in our population than we had predicted from an audit of our service so we had no reliable data on which to base a power calculation. Although we initially found a statistically significant increase in the risk of miscarriage with c < 4 mm, the CIs were high due to the small number of pregnancies implanted so close to a tubal ostium and after correction for multiple testing this was no longer significant. It is possible that a higher cut of 5 mm could be clinically relevant despite not reaching statistical significance., as over 50% of these patients miscarried.

In conclusion, we found that normally located early pregnancies implanted within 5 mm of a tubal ostium had an increased risk of miscarriage, though in our population this finding did not reach statistical significance. A validation study with a larger population is warranted to determine whether there is an optimum measurement for defining pregnancies at increased risk of miscarriage that could warrant the description of ‘angular’.

Original data can be requested where reasonable from the author and will then be made available.
